# Changing clinical characteristics of pediatric inpatients with pneumonia during COVID-19 pandamic: a retrospective study

**DOI:** 10.1186/s13052-024-01651-8

**Published:** 2024-04-23

**Authors:** Mengxue Chen, Yabing Zhou, Shengjie Jin, Shasha Bai, Xiaoyu Tang, Quanhua Liu, Liwei Wang, Ruoxu Ji, Haipei Liu, Wenwei Zhong, Yi Chen, Dingzhu Fang, Jianhua Zhang, Li Hua

**Affiliations:** 1 Department of Pediatric Pulmonology, Xin Hua Hospital, Shanghai Jiao Tong University School of Medicine, 1665 Kongjiang Road, 200092 Shanghai, China; 2 Department of Traditional Chinese Medicine, Xin Hua Hospital, Shanghai Jiao Tong University School of Medicine, Shanghai, China; 3 Department of Mathematics, Statistics and Insurance, Hang Seng University of Hong Kong, Hong Kong, China

**Keywords:** Characteristics, Children, COVID-19, Pneumonia

## Abstract

**Background:**

The COVID-19 pandemic have impacts on the prevalence of other pathogens and people’s social lifestyle. This study aimed to compare the pathogen, allergen and micronutrient characteristics of pediatric inpatients with pneumonia prior to and during the COVID-19 pandemic in a large tertiary hospital in Shanghai, China.

**Methods:**

Patients with pneumonia admitted to the Department of Pediatric Pulmonology of Xinhua Hospital between March-August 2019 and March-August 2020 were recruited. And clinical characteristics of the patients in 2019 were compared with those in 2020.

**Results:**

Hospitalizations for pneumonia decreased by 74% after the COVID-19 pandemic. For pathogens, virus, mycoplasma pneumoniae (MP) and mixed infection rates were all much lower in 2020 than those in 2019 (*P* < 0.01). Regarding allergens, compared with 2019, the positive rates of house dust mite, shrimp and crab were significantly higher in 2020 (*P* < 0.01). And for micronutrients, the levels of vitamin B2, B6, C and 25-hydroxyvitamin D (25(OH)D) in 2020 were observed to be significantly lower than those in 2019 (*P* < 0.05). For all the study participants, longer hospital stay (OR = 1.521, *P* = 0.000), milk allergy (OR = 6.552, *P* = 0.033) and calcium (Ca) insufficiency (OR = 12.048, *P* = 0.019) were identified as high-risk factors for severe pneumonia by multivariate analysis.

**Conclusions:**

The number of children hospitalized with pneumonia and incidence of common pathogen infections were both reduced, and that allergy and micronutrient status in children were also changed after the outbreak of the COVID-19 pandemic.

## Introduction

Pneumonia is the leading infectious cause of death amongst children worldwide. It accounts for more than 138 million new cases and almost one million deaths annually, mostly amongst children under 5 years old [1, 2]. Numerous pathogens, both viral and bacterial, are involved in pediatric pneumonia. Research showed a concomitant decrease in all-cause pneumonia during the COVID‐19 pandemic in China [3]. These changes might have come from the competition of severe acute respiratory syndrome coronavirus-2 (SARS-CoV-2) with other respiratory viruses, but they were more likely to have come from improvements in population hygiene practices to prevent SARS-CoV-2 transmission, such as wearing facemasks, paying attention to hand hygiene [4].

SARS-CoV-2 is the third zoonotic and highly pathogenic coronavirus to emerge in the twenty-first century [5]. And the virus has underwent frequent mutations and recombinations, yielding new variants that can cross the species barrier [6]. The World Health Organization (WHO) has identified five variants of concern as Alpha, Beta, Gamma, Delta, and the latest one named Omicron [7]. COVID-19 caused by SARS-CoV-2 emerged in December 2019 in Wuhan, Hubei Province, China [5]. On 11 March 2020, the WHO declared the COVID-19 pandemic. Since then, the number of globally confirmed COVID-19 cases has been increasing exponentially, which has engendered substantial health and economic burdens worldwide [8]. Governmental agencies of different countries worldwide have been widely promoting several measures to prevent the COVID-19 outbreak. Before the vaccination programme at the end of 2020, the fight against the COVID-19 epidemic mainly relied on preventive measures, including encouraging individual-level hygiene (wearing a mask outside, keeping social distance, disinfecting with alcohol and washing hands frequently) and community-level prevention measures (promotion of remote work and study, suspension of mass gathering) [9–12]. These non-pharmaceutical interventions (NPIs) not only decrease the spreading of the SARS-CoV-2, but also have impact on the prevalence of other pathogens [13–15]. Moreover, public health measures during COVID-19 have led to an unprecedented change in social lifestyle which might have effect on the allergen sensitization and micronutrient levels in population [16, 17].

In this context, our study aimed to compare the pathogen, allergen and micronutrient characteristics of pediatric inpatients with pneumonia prior to and during the COVID-19 pandemic in a large tertiary hospital in Shanghai, China.

## Methods

### Study participants

This was a retrospective study and conducted in Xinhua Hospital, a large tertiary hospital in Shanghai, China. The inclusion criteria for the study were: pneumonia patients admitted to the Department of Pediatric Pulmonology of Xinhua Hospital from March to August 2020 (during the COVID-19 pandemic when all students studied online at home and strengthened personal protection) and in the same period of 2019 (prior to the COVID-19 pandemic when all students studied at school); negative for COVID-19; and aged 3 months to 16 years. Children were diagnosed with pneumonia or severe pneumonia based on the criteria recommended by the 2011 pediatric community-acquired pneumonia guidelines [18]. Since the COVID-19 pandemic, all pneumonia patients were screened for COVID-19 using real-time reverse transcriptase polymerase chain reaction and each recruited child was confirmed negative for COVID-19. Patients with congenital abnormalities, immunodeficiency, metabolic disorders, chronic conditions, underlying lung diseases, airway foreign bodies, tuberculosis or nosocomial respiratory infections were excluded from this study.

Written informed consent was obtained from parents or legal guardians for all the subjects who are under 16. The study was approved by the Ethics Committee of Xinhua Hospital (approval number: Approval No. XHEC-D-2023-019), and conducted according to the principles in the Declaration of Helsinki.

### Data collection and specimen detection

The study participants’ clinical characteristics, including age, sex, length of stay, season of hospitalization, pathogen, allergen-specific IgE (sIgE) and micronutrient levels were collected from the electronic medical records.

Peripheral blood samples and respiratory specimens were obtained on admission by trained nurses following standard operating procedures. Pathogen detections were conducted, including: bacterial cultures prepared from secretions of lower respiratory tracts; direct immunofluorescence assay for the virus antibody tests, including respiratory syncytial virus (RSV), adenovirus (ADV), influenza virus (IV), parainfluenza virus (PIV) and coxsackie virus; and mycoplasma pneumonia (Mp) IgM tested with ELISA. Mp infection was defined as seropositivity of Mp IgM in the acute stage.

Five main allergens, namely house dust mite, milk, egg white, shrimp and crab were evaluated in this study. Serum sIgE was measured by ImmunoCAP, and concentration ≥ 0.35 IU/ml was considered as positive, otherwise, as negative [19].

The explored micronutrients in our study included vitamin A, B1, B2, B6, C, serum 25-hydroxyvitamin D (25(OH)D), calcium (Ca) and zinc (Zn). Vitamins were analyzed with electrochemical detection, while calcium and zink were determined with atomic absorption spectrometry.

### Statistical analysis

Clinical characteristics of the study participants prior to the COVID-19 pandemic were compared with those during the COVID-19 pandemic. All data were entered into the statistical package SPSS version 25.0 (SPSS Inc., Chicago, IL, USA). Continuous variables were expressed as median (interquartile range, IQR), and compared with Mann-Whitney U test. Categorical variables were presented as number (%) and analyzed using Chi-square test or Fisher’s exact test. Binary logistic regression analysis was used to examine the related factors of severe pneumonia in children. Two-sided *P* values ≤ 0.05 were considered statistically significant.

## Results

### Clinical characteristics of pediatric inpatients with pneumonia prior to and during the COVID-19 pandemic

**Fig. 1:**
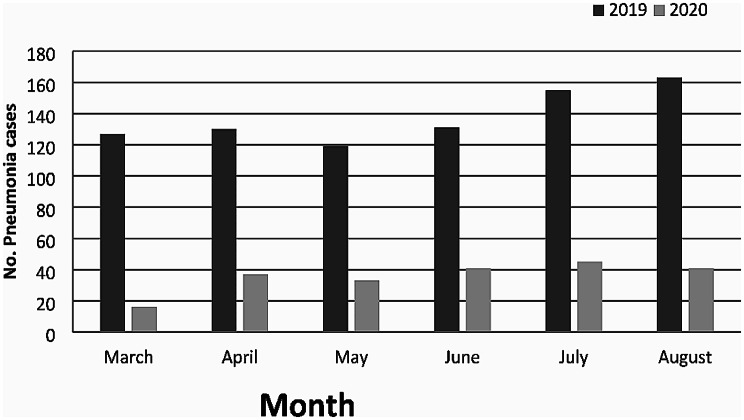
Monthly distribution of pediatric inpatients with pneumonia in Xinhua Hospital, Shanghai, China (2019–2020)

A total of 1038 children with pneumonia admitted to the Department of Pediatric Pulmonology of Xinhua Hospital were enrolled, including 825 cases occurred from March to August 2019 and 213 cases during the same period in 2020, which indicated a reduction of 74% in the number of pneumonia inpatients after the outbreak of the COVID-19 pandemic. Figure 1 depicts monthly distribution of these inpatients.

Clinical characteristics of the pneumonia inpatients prior to and during the COVID-19 pandemic were compared, as shown in Table 1. The median age of the patients in 2020 was much smaller than that in 2019 (*P* < 0.01), while the median hospital stay in 2020 was signifcantly longer than that in 2019 (*P* < 0.01). No differences were found in gender and season of hospitalization between the two years (*P* > 0.05).

**Table 1:** Clinical characteristics of pediatric inpatients with pneumonia prior to and during the COVID-19 pandemic

Characteristics	Prior to the COVID-19 pandemic (1 March 2019–31 August 2019)	During the COVID-19 pandemic (1 March 2020–31 August 2020)	P value^a^
Number of cases, n(%)	825(100.0)	213(100.0)	
Age, median(IQR), m	53(26.0–84.0)	26(9.0–52.0)	0.000*
Gender, n(%)			0.240
Male	451(54.7)	126(59.2)	
Female	374(45.3)	87(40.8)	
Hospital stay, median(IQR), d	6(5.0–8.0)	7(5.0–10.0)	0.000*
Season of hospitalization, n(%)			0.173
Spring(Mar.-May.)	376(45.6)	86(40.4)	
Summer(Jun.-Aug.)	449(54.4)	127(59.6)	
Severity, n(%)			0.242
Mild	782(94.8)	206(96.7)	
Severe	43(5.2)	7(3.3)	
Bacterial infections, n(%)	52(6.3)	21(9.9)	0.070
Moraxella catarrhalis	4(0.5)	2(0.9)	
Haemophilus influenzae	21(2.5)	2(0.9)	
Streptococcus pneumoniae	11(1.3)	4(1.9)	
Staphylococcus	11(1.3)	5(2.3)	
Acinetobacter baumannii	3(0.4)	1(0.5)	
Pseudomonas aeruginosa	2(0.2)	3(1.4)	
Klebsiella pneumoniae	0(0.0)	3(1.4)	
Enterobacter cloacae	0(0.0)	1(0.5)	
Viral infections, n(%)	143(17.3)	15(7.0)	0.000*
RSV	5(0.6)	0(0.0)	
ADV	34(4.1)	2(0.9)	
IV	55(6.7)	4(1.9)	
PIV	37(4.5)	2(0.9)	
Coxsackie virus	12(1.5)	7(3.3)	
Mp infection, n(%)	470(57.0)	83(39.0)	0.000*
Mixed infections, n(%)	134(16.2)	15(7.0)	0.001*
Bacteria + Bacteria	3(0.4)	1(0.5)	
Bacteria + Mp	17(2.1)	2(0.9)	
Bacteria + Viruses	3(0.4)	0(0.0)	
Bacteria + Mp + Viruses	5(0.6)	2(0.9)	
Viruses + Mp	106(12.8)	9(4.2)	
Viruses + Viruses	0(0.0)	1(0.5)	
Serum sIgE(IU/ml), n(%)			
House dust mite			0.000*
< 0.35	613(74.3)	86(40.4)	
≥ 0.35	109(13.2)	38(17.8)	
Milk			0.105
< 0.35	571(69.2)	90(42.3)	
≥ 0.35	151(18.3)	34(16.0)	
Egg white			0.192
< 0.35	646(78.3)	106(49.8)	
≥ 0.35	76(9.2)	18(8.5)	
Shrimp			0.000*
< 0.35	713(86.4)	115(54.0)	
≥ 0.35	9(1.1)	9(4.2)	
Crab			0.000*
< 0.35	715(86.7)	115(54.0)	
≥ 0.35	7(0.8)	9(4.2)	
Micronutrient levels			
Vitamin A(umol/l)			0.137
< 0.52	13(1.6)	4(1.9)	
0.52–2.2	466(56.5)	49(23.0)	
Vitamin B1(nmol/l )			0.939
< 50	21(2.5)	3(1.4)	
50–150	458(55.5)	50(23.5)	
Vitamin B2(ug/l)			0.044*
< 200	25(3.0)	7(3.3)	
≥ 200	454(55.0)	46(21.6)	
Vitamin B6(umol/l)			0.000*
< 14.6	1(0.1)	4(1.9)	
14.6–72.9	478(57.9)	49(23.0)	
Vitamin C(umol/l)			0.000*
< 34	48(5.8)	17(8.0)	
34–114	431(52.2)	36(16.9)	
Serum 25(OH)D(nmol/l)			0.000*
< 50	0(0.0)	9(4.2)	
≥ 50	478(57.9)	44(20.7)	
Zn(umol/l)			0.756
< 9.2	86(10.4)	10(4.7)	
9.2–22.9	348(42.2)	36(16.9)	
Ca(mmol/l)			0.100
< 2.0	30(3.6)	0(0.0)	
2.0-2.74	404(49.0)	46(21.6)	

IQR, interquartile range; RSV, respiratory syncytial virus; ADV, adenovirus; IV, influenza virus; PIV, parainfluenza virus; Mp, mycoplasma pneumoniae; sIgE, specific IgE; 25(OH)D, 25-hydroxyvitamin D; Zn, zink; Ca, Calcium

^a^*P* values for Mann-Whitney U Test, chi-square test or Fisher’s exact test

*P ≤ 0.05

For pathogens, both virus and MP infection rate were much lower during the COVID-19 pandemic than those prior to it (*P* < 0.01), whereas no significant difference was found in bacterial infection rate between the two years (*P* > 0.05). In virus infections, coxsackie virus was the most common, followed by IV, PIV and ADV in 2020, while IV was the most common virus, followed by PIV, ADV and coxsackie virus in 2019. Compared with 2019, the positive rates of RSV, ADV, IV and PIV were decreased in 2020. However, the positive rate of coxsackie virus was higher in 2020 than that in 2019. Mixed infections were signifcantly less frequent during the COVID-19 pandemic than those before the pandemic (*P* < 0.01). Co-infections with viruses and MP were the most common type of mixed infection in both 2019 and 2020.

Regarding allergens, compared with 2019, the positive rates of house dust mite, shrimp and crab were significantly higher in 2020 (*P* < 0.01). And for micronutrients, the levels of vitamin B2, B6, C and 25(OH)D in 2020 were observed to be significantly lower than those in 2019 (*P* < 0.05).

### Clinical characteristics of pediatric inpatients with severe pneumonia prior to and during the COVID-19 pandemic

Among the participants, 5.2% (43/825) in 2019 and 3.3% (7/213) in 2020 were diagnosed with severe pneumonia respectively, showing no difference between the two years (*P* > 0.05). Table 2 showed the clinical characteristics of the children with severe pneumonia, and significant differences were found in age and vitamin B6 status between the two years (*P* ≤ 0.05). Severe pneumonia inpatients in 2020 with median age of 6 months were much younger than those in 2019 with median age of 35 months. The level of vitamin B6 in 2020 was lower than that in 2019. No differences were found in other characteristics between the two years (*P* > 0.05).

**Table 2:** Clinical characteristics of pediatric inpatients with severe pneumonia prior to and during the COVID-19 pandemic

Characteristics	Prior to the COVID-19 pandemic (1 March 2019–31 August 2019)	During the COVID-19 pandemic (1 March 2020–31 August 2020)	P value^a^
Number of cases, n (%)	43(100.0)	7(100.0)	
Age, median (IQR), m	35(17.0–67.0)	6(5.0–14.0)	0.003*
Gender, n (%)			0.819
Male	24(55.8)	3(42.9)	
Female	19(44.2)	4(57.1)	
Hospital stay, median (IQR), d	11(9.0–14.0)	12(9.0–29.0)	0.528
Season of hospitalization, n (%)			0.405
Spring (Mar.-May.)	29(67.4)	3(42.9)	
Summer(Jun.-Aug.)	14(32.6)	4(57.1)	
Bacterial infections, n (%)			0.541
No	38(88.4)	5(71.4)	
Yes	5(11.6)	2(28.6)	
Viral infections, n (%)			0.308
No	26(60.5)	6(85.7)	
Yes	10(23.3)	0(0.0)	
Mp infection, n (%)			0.448
No	17(39.5)	5(71.4)	
Yes	19(44.2)	2(28.6)	
Mixed infections, n (%)			0.943
No	26(60.5)	5(71.4)	
Yes	10(23.3)	1(14.3)	
Serum sIgE(IU/ml), n (%)			
House dust mite			1.000
< 0.35	23(53.5)	1(14.3)	
≥ 0.35	8(18.6)	1(14.3)	
Milk			1.000
< 0.35	21(48.8)	2(28.6)	
≥ 0.35	10(23.3)	0(0.0)	
Egg white			1.000
< 0.35	27(62.8)	2(28.6)	
≥ 0.35	4(9.3)	0(0.0)	
Shrimp			1.000
< 0.35	30(69.8)	2(28.6)	
≥ 0.35	1(2.3)	0(0.0)	
Crab			1.000
< 0.35	30(69.8)	2(28.6)	
≥ 0.35	1(2.3)	0(0.0)	
Micronutrient levels			
Vitamin A(umol/l)			1.000
< 0.52	2(4.7)	0(0.0)	
0.52–2.2	17(39.5)	1(14.3)	
Vitamin B1(nmol/l )			-
< 50	0(0.0)	0(0.0)	
50–150	19(44.2)	1(14.3)	
Vitamin B2(ug/l)			1.000
< 200	2(4.7)	0(0.0)	
≥ 200	17(39.5)	1(14.3)	
Vitamin B6(umol/l)			0.050*
< 14.6	0(0.0)	1(14.3)	
14.6–72.9	19(44.2)	0(0.0)	
Vitamin C(umol/l)			0.150
< 34	2(4.7)	1(14.3)	
34–114	17(39.5)	0(0.0)	
Serum 25(OH)D(nmol/l)			-
< 50	0(0.0)	0(0.0)	
≥ 50	19(44.2)	1(14.3)	
Zn(umol/l)			0.429
< 9.2	12(27.9)	0(0.0)	
9.2–22.9	8(18.6)	1(14.3)	
Ca(mmol/l)			1.000
< 2.0	9(20.9)	0(0.0)	
2.0-2.74	11(25.6)	1(14.3)	

IQR, interquartile range; Mp, mycoplasma pneumoniae; sIgE, specific IgE; 25(OH)D, 25-hydroxyvitamin D; Zn, zink; Ca, Calcium

^a^*P* values for Mann-Whitney U Test, chi-square test or Fisher’s exact test

*P ≤ 0.05

### Related factors of severe pneumonia in pediatric inpatients prior to and during the COVID-19 pandemic by univariate analysis

Through univariate analysis, related factors of severe pneumonia were explored among the patients prior to and during the COVID-19 pandemic respectively. As shown in Table 3, younger age, longer hospital stay, hospitalization in spring and insufficiency of Ca and Zn had significant correlations with severe pneumonia among the children in 2019 (*P* < 0.05). However, in 2020, only younger age and longer hospital stay were found associated with severe pneumonia (*P* < 0.05).

**Table 3:** Related factors of severe pneumonia in pediatric inpatients prior to and during the COVID-19 pandemic by univariate analysis

Characteristics	Prior to the COVID-19 pandemic (1 March 2019–31 August 2019)			During the COVID-19 pandemic (1 March 2020–31 August 2020)		
	non-severe group	Severe group	P value^a^	non-severe group	Severe group	P value^a^
Number of cases, n (%)	782(100.0)	43(100.0)		206(100.0)	7(100.0)	
Age, median (IQR), m	54(26.0–85.0)	35(17.0–67.0)	0.036*	28(10.0–52.0)	6(5.0–14.0)	0.038*
Gender, n (%)			0.877			0.616
Male	427(54.6)	24(55.8)		123(59.7)	3(42.9)	
Female	355(45.4)	19(44.2)		83(40.3)	4(57.1)	
Hospital stay, median (IQR), d	6(5.0–7.0)	11(9.0–14.0)	0.000*	7(5.0–9.0)	12(9.0–29.0)	0.002*
Season of hospitalization, n (%)			0.003*			1.000
Spring (Mar.-May.)	347(44.4)	29(67.4)		83(40.3)	3(42.9)	
Summer(Jun.-Aug.)	435(55.6)	14(32.6)		123(59.7)	4(57.1)	
Bacterial infections, n (%)			0.249			0.296
No	735(94.0)	38(88.4)		187(90.8)	5(71.4)	
Yes	47(6.0)	5(11.6)		19(9.2)	2(28.6)	
Viral infections, n (%)			0.121			1.000
No	623(79.7)	26(60.5)		179(86.9)	6(85.7)	
Yes	133(17.0)	10(23.3)		15(7.3)	0(0.0)	
Mp infection, n (%)			0.412			0.785
No	305(39.0)	17(39.5)		116(56.3)	5(71.4)	
Yes	451(57.7)	19(44.2)		81(39.3)	2(28.6)	
Mixed infections, n (%)			0.075			0.940
No	632(80.8)	26(60.5)		179(86.9)	5(71.4)	
Yes	124(15.9)	10(23.3)		14(6.8)	1(14.3)	
Serum sIgE(IU/ml), n (%)						
House dust mite			0.148			1.000
< 0.35	590(75.4)	23(53.5)		85(41.3)	1(14.3)	
≥ 0.35	101(12.9)	8(18.6)		37(18.0)	1(14.3)	
Milk			0.112			1.000
< 0.35	550(70.3)	21(48.8)		88(42.7)	2(28.6)	
≥ 0.35	141(18.0)	10(23.3)		34(16.5)	0(0.0)	
Egg white			0.887			1.000
< 0.35	619(79.2)	27(62.8)		104(50.5)	2(28.6)	
≥ 0.35	72(9.2)	4(9.3)		18(8.7)	0(0.0)	
Shrimp			0.851			1.000
< 0.35	683(87.3)	30(69.8)		113(54.9)	2(28.6)	
≥ 0.35	8(1.0)	1(2.3)		9(4.4)	0(0.0)	
Crab			0.709			1.000
< 0.35	685(87.6)	30(69.8)		113(54.9)	2(28.6)	
≥ 0.35	6(0.8)	1(2.3)		9(4.4)	0(0.0)	
Micronutrient levels						
Vitamin A(umol/l)			0.156			1.000
< 0.52	11(1.4)	2(4.7)		4(1.9)	0(0.0)	
0.52–2.2	449(57.4)	17(39.5)		48(23.3)	1(14.3)	
Vitamin B1(nmol/l )			1.000			1.000
< 50	21(2.7)	0(0.0)		3(1.5)	0(0.0)	
50–150	439(56.1)	19(44.2)		49(23.8)	1(14.3)	
Vitamin B2(ug/l)			0.593			1.000
< 200	23(2.9)	2(4.7)		7(3.4)	0(0.0)	
≥ 200	437(55.9)	17(39.5)		45(21.8)	1(14.3)	
Vitamin B6(umol/l)			1.000			0.075
< 14.6	1(0.1)	0(0.0)		3(1.5)	1(14.3)	
14.6–72.9	459(58.7)	19(44.2)		49(23.8)	0(0.0)	
Vitamin C(umol/l)			1.000			0.321
< 34	46(5.9)	2(4.7)		16(7.8)	1(14.3)	
34–114	414(52.9)	17(39.5)		36(17.5)	0(0.0)	
Serum 25(OH)D(nmol/l)			-			1.000
< 50	0(0.0)	0(0.0)		9(4.4)	0(0.0)	
≥ 50	459(58.7)	19(44.2)		43(20.9)	1(14.3)	
Zn(umol/l)			0.000*			1.000
< 9.2	74(9.5)	12(27.9)		10(4.9)	0(0.0)	
9.2–22.9	340(43.5)	8(18.6)		35(17.0)	1(14.3)	
Ca(mmol/l)			0.000*			-
< 2.0	21(2.7)	9(20.9)		0(0.0)	0(0.0)	
2.0-2.74	393(50.3)	11(25.6)		45(21.8)	1(14.3)	

IQR, interquartile range; Mp, mycoplasma pneumoniae; sIgE, specific IgE; 25(OH)D, 25-hydroxyvitamin D; Zn, zink; Ca, Calcium

^a^*P* values for Mann-Whitney U Test, chi-square test or Fisher’s exact test

*P ≤ 0.05

### Related factors of severe pneumonia in pediatric inpatients by multivariate analysis

Binary logistic regression analysis was used to examine risk factors of severe pneumonia among all the children. Table 4 showed that longer hospital stay (OR = 1.521, *P* = 0.000), milk allergy (OR = 6.552, *P* = 0.033) and Ca insufficiency (OR = 12.048, *P* = 0.019) were high-risk factors for severe pneumonia in the pediatric inpatients, after adjusting for other factors.

**Table 4:** Related factors of severe pneumonia in pediatric inpatients by multivariate analysis

Characteristics	non-severe group	Severe group	cOR	95%CI	P value^a^	aOR	95%CI	P value^b^
Number of cases, n (%)	988(100.0)	50(100.0)						
Age, median (IQR), m	48(21.0–82.0)	32(14.0–65.0)	0.990	(0.981,0.999)	0.022*	0.992	(0.967,1.018)	0.558
Gender, n (%)					0.817			0.583
Male	550(55.7)	27(54.0)	1.000			1.000		
Female	438(44.3)	23(46.0)	1.070	(0.605,1.892)		1.548	(0.325,7.386)	
Hospital stay, median (IQR), d	6(5.0–8.0)	12(9.0–14.0)	1.434	(1.324,1.553)	0.000*	1.521	(1.241,1.866)	0.000*
Season of hospitalization, n (%)					0.006*			0.299
Spring (Mar.-May.)	430(43.5)	32(64.0)	1.000			1.000		
Summer(Jun.-Aug.)	558(56.5)	18(36.0)	0.433	(0.240,0.783)		0.359	(0.052,2.479)	
Period, n (%)					0.246			0.998
Pre-COVID-19	782(79.1)	43(86.0)	1.000			1.000		
COVID-19	206(20.9)	7(14.0)	0.618	(0.274,1.394)		0.000	0.000	
Bacterial infections, n (%)					0.054			0.585
No	922(93.3)	43(86.0)	1.000			1.000		
Yes	66(6.7)	7(14.0)	2.274	(0.985,5.252)		2.111	(0.144,30.932)	
Viral infections, n (%)					0.158			0.273
No	802(81.2)	32(64.0)	1.000			1.000		
Yes	148(15.0)	10(20.0)	1.693	(0.815,3.519)		3.762	(0.353,40.114)	
Mp infection, n (%)					0.369			0.242
No	421(42.6)	22(44.0)	1.000			1.000		
Yes	532(53.8)	21(42.0)	0.755	(0.410,1.392)		0.294	(0.038,2.284)	
Mixed infections, n (%)					0.043*			0.592
No	811(82.1)	31(62.0)	1.000			1.000		
Yes	138(14.0)	11(22.0)	2.085	(1.024,4.246)		2.249	(0.116,43.534)	
Serum sIgE(IU/ml), n (%)								
House dust mite					0.131			0.499
< 0.35	675(68.3)	24(48.0)	1.000			1.000		
≥ 0.35	138(14.0)	9(18.0)	1.834	(0.834,4.032)		0.512	(0.074,3.564)	
Milk					0.235			0.033*
< 0.35	638(64.6)	23(46.0)	1.000			1.000		
≥ 0.35	175(17.7)	10(20.0)	1.585	(0.741,3.393)		6.552	(1.162,36.936)	
Egg white					0.851			0.261
< 0.35	723(73.2)	29(58.0)	1.000			1.000		
≥ 0.35	90(9.1)	4(8.0)	1.108	(0.381,3.224)		2.916	(0.450,18.890)	
Shrimp					0.716			1.000
< 0.35	796(80.6)	32(64.0)	1.000			1.000		
≥ 0.35	17(1.7)	1(2.0)	1.463	(0.189,11.338)		0.000	0.000	
Crab					0.628			1.000
< 0.35	798(80.8)	32(64.0)	1.000			1.000		
≥ 0.35	15(1.5)	1(2.0)	1.662	(0.213,12.977)		0.127	0.000	
Micronutrient levels								
Vitamin A(umol/l)					0.099			0.105
0.52–2.2	497(50.3)	18(36.0)	1.000			1.000		
< 0.52	15(1.5)	2(4.0)	3.681	(0.783,17.320)		15.604	(0.563,432.153)	
Vitamin B1(nmol/l )					0.998			0.998
50–150	488(49.4)	20(40.0)	1.000			1.000		
< 50	24(2.4)	0(0.0)	0.000	0.000		0.000	0.000	
Vitamin B2(ug/l)					0.451			0.904
≥ 200	482(48.8)	18(36.0)	1.000			1.000		
< 200	30(3.0)	2(4.0)	1.785	(0.396,8.054)		1.264	(0.029,55.840)	
Vitamin B6(umol/l)					0.096			0.999
14.6–72.9	508(51.4)	19(38.0)	1.000			1.000		
< 14.6	4(0.4)	1(2.0)	6.684	(0.713,62.702)		0.000	0.000	
Vitamin C(umol/l)					0.699			0.902
34–114	450(45.5)	17(34.0)	1.000			1.000		
< 34	62(6.3)	3(6.0)	1.281	(0.365,4.496)		1.175	(0.090,15.388)	
Serum 25(OH)D(nmol/l)					0.999			1.000
≥ 50	502(50.8)	20(40.0)	1.000			1.000		
< 50	9(0.9)	0(0.0)	0.000	0.000		0.293	0.000	
Zn(umol/l)					0.000*			0.570
9.2–22.9	375(38.0)	9(18.0)	1.000			1.000		
< 9.2	84(8.5)	12(24.0)	5.952	(2.430,14.583)		1.760	(0.250,12.386)	
Ca(mmol/l)					0.000*			0.019*
2.0-2.74	438(44.3)	12(24.0)	1.000			1.000		
< 2.0	21(2.1)	9(18.0)	15.643	(15.937,41.217)		12.103	(1.510,97.011)	

cOR, crude odds ratio; aOR, adjusted odds ratio; IQR, interquartile range; COVID-19, coronavirus disease 2019; Mp, mycoplasma pneumoniae; sIgE, specific IgE; 25(OH)D, 25-hydroxyvitamin D; Zn, zink; Ca, Calcium

^a^*P* values for Binary logistic Regression, not adjusted for other factors

^b^*P* values for Binary logistic Regression, adjusted for other factors

*P ≤ 0.05

## Discussion

We found that between March-August 2020, the number of children hospitalized with pneumonia at Xinhua Hospital was decreased by 74% compared with the same period of 2019. Recent studies have witnessed a significant reduction in the numbers of pediatric admissions in 2020 in many other countries, including France, the United States and Morocco [20, 21]. This was due to the recommendations imposed at the beginning of the pandemic to avoid access to hospital, except in cases of real need, and also to the public fear of SARS-CoV‐2 infection in the hospital [22]. Additionly, the change in lifestyle during the COVID-19 pandemic might also have reduced the onset of respiratory viral diseases by preventing much person-to-person disease transmission [23, 24]. In other words, this reduction could be considered as an unexpected positive consequence of the NPIs taken during the COVID-19 pandemic, which may have led to a decrease in morbidity and healthcare costs of the infectious diseases [25].

For pathogens, our results showed that viral, Mp and mixed infection rate was reduced respectively in 2020 in children with pneumonia, which was similar to Pengcheng Liu et al’s, Ying Zhang et al’s and Huang QS et al’s reports, possibly because of the use of stringent NPIs such as lockdown and border closures in 2020 [25–27]. However, in our study, the detection of coxsackie virus increased compared with 2019. The result is in line with the study of Andrew Po-Liang Chen et al’s in Taiwan [28]. Since coxsackie virus is a non-enveloped virus, it might be inherently less susceptible to inactivation by ethanol-containing disinfectant [29]. This viral property might hamper the preventive effectiveness using routine hand disinfectants. Furthermore, Leung et al’s randomized control trial suggests that face mask is more effective in filtering out enveloped viruses than non-enveloped viruses [30]. Consequently, chloride- and hydrogen peroxide-based products should be added for comprehensive infection control. In addition, it is important that coxsackie virus should continue to be monitored diligently in children during the COVID-19 pandemic. And the diseases caused by coxsackie virus should be taken seriously such as hand-foot-mouth disease (HFMD) or herpangina.

Change in lifestyle could trigger the change of people’s susceptibility to various allergens [16]. Our study showed that the positive rates of allergens, including house-dust mite, shrimp and crab in 2020 were significantly higher than those in 2019 in children with pneumonia. The increasing positive rate of house dust mite during the COVID-19 pandemic might be because people stayed at home for a long time with poor ventilation. Shanghai has a humid subtropical monsoon climate. The lack of ventilation led to long-term high relative humidity and lack of sunshine in the house, which were more conducive to the accumulation and reproduction of dust mites in carpets, pillows, and mattresses [31, 32]. Moreover, house dust mite allergen, *Der p1*, exerts several key activities on the airway mucosa [33]. Such molecules have been shown to favor oxidative stress of the respiratory mucosa and, thus, to exacerbate inflammatory conditions such as asthma or allergic rhinitis [34]. Regarding shrimp and crab allergens, the reason would be that people’s eating habits had been changed during the pandemic and it also might be related to the co-sensitization with the increasing positive rate of house dust mite [35]. Therefore, during home isolation, our room should be fully ventilated, and the sheets and blankets should be washed frequently.

Our study found that hospitalized patients with pneumonia in children presented the lower levels of vitamin B2, B6, C and 25(OH)D in 2020 than those in 2019. The possible reason would be that the COVID-19 lockdown has affected the dietary habits and nutritional patterns. It has been reported that the dietary patterns of Chinese people during the COVID-19 lockdown changed, showing a decrease in the frequency of intake of fresh vegetables and fruit, rice, poultry, meat, and soybean products [36]. However, certain nutrients such as vitamin A, vitaminB2, vitamin B6, vitamin B12, vitamin D, vitamin C, and the minerals Ca and Zn, are important for proper immune function [37]. Deficiencies and a suboptimal nutritional status of these micronutrients could potentially favor the spread of diseases by reducing resistance to infection and reinfection [17]. For example, the vitamin D receptor is expressed in almost all types of cells of the immune system and the correct immune system function will depend on the correct bioavailability of vitamin D from these cells [38]. For vitamin C, it also has roles in several aspects of immunity, including supporting leucocytes migration to sites of infection, phagocytosis and bacterial killing, natural killer cell activity and antibody production [39]. To date, despite the existence of several vaccines in motion to deal with the SARS-CoV-2, the global population must learn to live for a longer time with the virus among us [17]. Therefore, we should keep a healthy and balanced diet in our daily life to strengthen our natural defense system.

There were some limitations in this retrospective study. First, our report was a single-center study, and we only enrolled pneumonia inpatients in our clinical center between March-August 2020 when all students studied online at home and stringent public health measures were adopted during the COVID-19 pandemic. From September 2020, students went back to school. As the control, pneumonia inpatients in the same period of 2019 were recruited prior to the COVID-19 pandemic. Our results were based on a small number of pneumonia cases, and thus they should be interpreted with caution and confirmed in future studies. Second, the identification of the pathogens responsible for pneumonia remains challenging, particularly when mixed infections occur. A proportion of children in this study had no proven causal pathogen. The low detection rate of bacteria and viruses in our study may be due to the fact that some inpatients received antibiotic therapy in outpatient department. The use of next-generation sequencing for pathogen detection could be a useful additional method. Third, only five main allergens were analyzed in this study. Finally, the effects of micronutrient supplementation was not taken into account due to lack of information in this aspect.

## Conclusions

The data in this study suggest that number of children hospitalized with pneumonia and incidence of common pathogen infections were both reduced, and that allergy and micronutrient status in children were also changed after the outbreak of the COVID-19 pandemic and adoption of stringent public health measures.

## Data Availability

The datasets generated during and/or analysed during the current study are available from the corresponding author on reasonable request.
